# Competitive cobalt for zinc substitution in mammalian methionine sulfoxide reductase B1 overexpressed in *E. coli*: structural and functional insight

**DOI:** 10.1007/s00775-013-1064-7

**Published:** 2013-11-24

**Authors:** Elena Shumilina, Olena Dobrovolska, Rebecca Del Conte, Henrik Waldal Holen, Alexander Dikiy

**Affiliations:** 1 Department of Biotechnology, Norwegian University of Science and Technology, 7491 Trondheim, Norway; 2 CERM - Department of Chemistry, University of Florence, 50019 Sesto Fiorentino, Florence Italy

**Keywords:** Cell metabolism, Metal homeostasis, Metalloenzymes, Protein–metal ion interaction, NMR

## Abstract

Expression of the mammalian enzyme methionine sulfoxide reductase B1 (MsrB1) in *Escherichia coli* growing in cobalt-containing media resulted in the reproducible appearance of the stable cobalt-containing protein MsrB1-Co. NMR studies and biocomputing using the programs AnisoFit and Amber allowed us to generate a structure of MsrB1-Co sharing the overall fold with the native zinc-containing protein MsrB1-Zn. Our data suggest that the N-terminus containing resolving cysteine tends to be closer to the protein’s catalytic center than was previously reported. It is argued that this proximity supports the proposed catalytic mechanism and ensures high catalytic efficiency of MsrB1. Functional studies showed that both MsrB1-Zn and MsrB1-Co exhibit similar levels of activity, in agreement with the structural studies performed. The proposed metal ion substitution approach may have a methodological significance in determining whether methionine sulfoxide reductase B proteins contain a metal ion.

**Electronic supplementary material:**

The online version of this article (doi:10.1007/s00775-013-1064-7) contains supplementary material, which is available to authorized users.

## Introduction

Metal-containing proteins constitute about one third of all proteins found in nature [1, 2]. Metal atoms are often essential to their function, structure, and stability. The occurrence of these metals is quite different. Whereas magnesium, zinc, and iron are the most widespread metals, cobalt is found almost exclusively in B_12_-dependent enzymes [2]. Biometals are usually covalently bound to the nitrogen, oxygen, and sulfur atoms of polypeptide’s amino acid side chains [3]. The relative stability of metal complexes with endogenous protein ligands follows the Irving–Williams series: Ca^2+^ < Mg^2+^ < Mn^2+^ < Fe^2+^ < Co^2+^ < Ni^2+^ < Cu^2+^ > Zn^2+^ [4].

Zinc ion is one of the most important trace metal ions in living organisms and is an essential cofactor in many enzymes and proteins [1, 3, 5, 6]. It is possible to distinguish three types of zinc binding sites in proteins: catalytic, co-catalytic, and structural [7–10]. Structural Zn^2+^ sites have four protein ligands and no bound water molecule [8]. Four cysteines or two histidines in combination with two cysteines are common Zn^2+^ ligands in these types of sites. Mammalian methionine sulfoxide reductase B1 (MsrB1), the subject of the present study, contains a structural Zn^2+^ ion [11–14].

MsrB1 belongs to a family of redox repairing enzymes, methionine sulfoxide reductases (Msrs), that reduce both free and protein-bound methionine sulfoxide back to methionine [13, 15–18]. Two distinct subclasses constitute Msrs: MsrA and MsrB. MsrA is specific for reduction of methionine (*S*)-sulfoxide, whereas MsrB catalyzes the reduction of methionine (*R*)-sulfoxide. Mammals contain three MsrB subgroups—MsrB1, MsrB2, and MsrB3—having different cellular localization [12]. MsrB1 is a cytosolic and nuclear protein, MsrB2 is a mitochondrial protein, and MsrB3 occurs in the endoplasmic reticulum and mitochondria. In mammals, MsrB1 is a unique seleno-containing protein within the MsrB subclass, containing selenocysteine (Sec) in its active site. Sec is a rare amino acid that is co-translationally incorporated into proteins by UGA codons. Selenoproteins (i.e., Sec-containing proteins) are found in all three domains of life. Among the functionally characterized selenoproteins, most are oxidoreductases. The most striking characteristics provided by Sec in selenoenzymes is higher catalytic efficiency of these enzymes compared with their cysteine homologs [12, 19, 20].

The three-dimensional structure of mammalian MsrB1 has recently been determined by high-resolution NMR spectroscopy [21] and X-ray crystallography [Protein Data Bank (PDB) ID 3MAO]. The bioinformatics analysis [12] and the NMR data [21] revealed that MsrB1 contains a Zn^2+^ ion coordinated by four cysteine residues.

Cobalt ion is considered an excellent structural and functional model for studies of a protein’s zinc binding sites. Co^2+^–polypeptide complexes exhibit *d* → *d* transitions that absorb energy in the visible region of the electromagnetic spectrum, as well as charge transfer transitions in the UV region, whereas Zn^2+^ complexes are spectroscopically “silent.” At the same time, the coordination chemistry of Co^2+^ is very similar to that of Zn^2+^, and the two ions have almost identical radii [22–24]. Indeed, the ability of Co^2+^ ion to substitute for Zn^2+^ ion in proteins was reported elsewhere and has been used to study active sites of some naturally occurring zinc enzymes [25–27].

Generally, the substitution of Co^2+^ for Zn^2+^ in the proteins was performed by either displacement of the original metal ion (preparation of the apoprotein followed by addition of Co^2+^ ion) or by biosynthetic incorporation, which involves growing the host organism in a Co^2+^-supplemented and Zn^2+^-deficient medium [28–33]. There are only a few examples of in vivo substitution of Co^2+^ for Zn^2+^ ion in proteins [34, 35].

Our data indicate that Co^2+^ ion can competitively substitute Zn^2+^ ion in the mammalian MsrB1 protein when the latter is expressed in *Escherichia coli* growing in cobalt-supplemented medium. The structural and functional properties of Zn^2+^ and Co^2+^ MsrB1 derivatives, as indicated by our studies, are rather similar. A paramagnetic NMR study of the cobalt-containing MsrB1 (MsrB1-Co) derivative supports and complements the proposed MsrB1 catalytic mechanism, revealing that the mobile N-terminus is not fully unstructured, and tends to be in the vicinity of the protein catalytic center. Given the significant roles played by MsrB1 proteins in antioxidant defense, the possibility of cobalt–zinc substitution in MsrB1 is of interest, raising the question of the biological significance of the metal ion replacement.

## Materials and methods

### Microorganisms and plasmids

The genes of C-terminal and N-terminal (containing thrombin cleavage site) His-tagged mouse Sec95Cys MsrB1 (hereafter MsrB1), cloned into pET21 and pET28a expression vectors, were kindly provided by V. Gladyshev’s group and H.Y. Kim’s group, respectively. These plasmids were introduced into competent *E. coli* strain ER2566 (New England Biolabs) and strain BL21 (DE3) (Novagen) cells, correspondingly, using the standard protocol [36].

### Expression and purification of isotopically enriched MsrB1

*E. coli* cells carrying the appropriate plasmid were grown in M9 medium containing 99 % enriched (^15^NH_4_)_2_SO_4_ and 98 % enriched [^13^C_6_]glucose as well as 10 μM ZnSO_4_ and/or 10 μM CoCl_2_. Proteins were purified to homogeneity and their purity was examined using gel electrophoresis.

### Inhibition by CoCl_2_

*E. coli* cells (ER2566) carrying the pET21-MsrB1 plasmid were grown both in LB medium supplemented with 50 μM CoCl_2_, and in M9 medium containing a constant concentration of ZnSO_4_ (10 μM) and different concentrations of CoCl_2_ (2, 10, 50, 100, and 150 μM). Insertion of Co^2+^ ion into MsrB1 was then monitored using UV–visible and NMR spectroscopies.

### His-tag cleavage

A sample of cobalt-containing MsrB1 obtained from the pET28a vector, allowing the His-tag cleavage, was exchanged into thrombin cleavage buffer (50 mM sodium phosphate, 20 mM sodium chloride, pH 8.0). The His-tag was cleaved by thrombin (Novagen) (1 μl of thrombin was added to 1 mM MsrB1) by stirring for 3 h at room temperature. Thrombin and the His-tag were subsequently removed following the procedure provided by the supplier.

### NMR spectroscopy

The NMR samples contained 0.5 mM MsrB1 in NMR buffer (10 mM phosphate, pH 5.5, 10 mM NaCl, 5 mM β-mercaptoethanol) in 90 % H_2_O/10 % D_2_O. NMR spectra were recorded at 298 K with a Bruker Avance 600-MHz spectrometer equipped with 5-mm *z*-gradient TXI (H/C/N) cryoprobe at the NMR center of the Faculty of Natural Sciences and Technology of the Norwegian University of Science and Technology in Trondheim, and with a 600-MHz spectrometer equipped with an ^1^H-selective high-power probe (5 mm) at the CERM magnetic resonance center at the University of Florence. Proton chemical shifts were referenced to tetramethylsilane, and ^15^N and ^13^C chemical shifts were referenced indirectly to liquid ammonia and 3-(trimethylsilyl)-1-propanesulfonic acid sodium salt, correspondingly, on the basis of the absolute frequency ratios.

Two-dimensional ^15^N–^1^H heteronuclear single-quantum coherence (HSQC) spectra of MsrB1-Co and zinc-containing MsrB1 (MsrB1-Zn) proteins were compared and analyzed using TopSpin 2.1 (Bruker).

^1^H, ^15^N, and ^13^C backbone resonance assignments for MsrB1-Co were achieved using ^1^H–^15^N HSQC and ^1^H–^13^C HSQC, HNCA, HNCO, CACB(CO)NH, CACBNH, HBHANH, and HBHA(CO)NH NMR spectra. ^1^H and ^13^C side-chain assignment was achieved using the HCCH total correlation spectroscopy NMR spectrum and was used to confirm the backbone assignment obtained. The spectra were acquired using pulse sequences from the standard pulse sequence library. The NMR data were processed with Bruker TopSpin 2.1.

### Calculation of magnetic susceptibility anisotropy values

On the basis of the assignment of MsrB1-Co obtained and the available assignment of native MsrB1-Zn (Biological Magnetic Resonance Data Bank entry 15193), the calculation of pseudocontact shifts for HN and N atoms, which belong to the residues not directly bound to cobalt ion and, therefore, lack the chemical shift contact contribution, was performed with the following equation:

$$\delta^{\text{PCS}} = \delta^{\text{OBS}} - \delta^{\text{DIA}} ,$$

where *δ* ^PCS^ is the pseudocontact shift, *δ* ^OBS^ is the observed chemical shift, and *δ* ^DIA^ is the diamagnetic chemical shift.

Working with the hypothesis that Co^2+^ ion replaces Zn^2+^ in MsrB1, leading to little structural changes, we calculated the interatomic distances from Zn^2+^ to backbone HN and N atoms using the available MsrB1-Zn structure (PDB ID 2KV1) [21], and these distances were used as theoretical distances from Co^2+^ to the same atoms. A plot of pseudocontact shifts versus theoretical distances was built and used to evaluate the working hypothesis, check the assignments, and predict the chemical shifts for backbone HN and N atoms of the unassigned residues.

For further calculations three conformers with the lowest target function and the average conformer belonging to the MsrB1-Zn structure family (PDB ID 2KV1) were chosen. These structural data together with the pseudocontact shifts obtained were used for magnetic susceptibility anisotropy calculations with the aid of the program AnisoFit^1^ (previously known as Fantasia [37]) according to the following equation [38]:

1 $$\delta^{\text{PCS}} = \frac{1}{{12\pi r^{3} }}\left[ {\varDelta \chi_{\text{ax}}^{\text{para}} (3\cos^{2} \vartheta - 1) + \frac{3}{2}\varDelta \chi_{\text{rh}}^{\text{para}} (\sin^{2} \vartheta \cos 2\phi )} \right],$$

where $\varDelta \chi_{\text{ax}}^{\text{para}}$ and $\varDelta \chi_{\text{rh}}^{\text{para}}$ are the axial and rhombic anisotropies of the magnetic susceptibility tensor, $\vartheta$ and $\phi$ are cylindrical coordinates of the position vector of a proton, and *r* is the distance between the paramagnetic center and the proton. The result was optimized through the minimization of the third conformer, which was the most suitably fitted to the data obtained, by the program Amber 10 using the experimental pseudocontact shifts [39, 40]. The final magnetic susceptibility anisotropy values were obtained with the program AnisoFit over the minimized conformer using the We-NMR portals [41].

### One-dimensional ^1^H paramagnetic spectra

Proton NMR spectra over a broad spectral window (around 900 ppm) were acquired using a water presaturation pulse sequence at 600 MHz and a ^1^H-selective probe. One-dimensional nuclear Overhauser effect (NOE) experiments were performed by saturating some hyperfine-shifted and well-resolved signals. These experiments were recorded using a previously reported procedure [42, 43]. The residual water signal was suppressed by applying the SUPERWEFT pulse sequence 180°-*t*-90°-AQ, where the recycle time was 50 ms and *t* was adjusted to values between 50 and 30 ms to minimize the water signal. All spectra were acquired in deuterated buffer.

### UV–visible spectroscopy

UV–visible absorption spectra were recorded with an Ultrospec 2000 UV–visible spectrophotometer (Pharmacia Biotech) using quartz cuvettes with 1-cm path length. Wavescan version 1.02 was used.

### Removal of metal from MsrB1-Co and MsrB1-Zn

Samples of MsrB1-Co and MsrB1-Zn were exchanged into 50 mM phosphate buffer, containing 30 mM NaCl, 5 mM β-mercaptoethanol, at pH 5.0 and pH 7.0. Then, 4 ml of each sample was dialyzed against 2 l of the same buffer containing two chelating agents—5 mM ethylene glycol bis(2-aminoethyl ether)-*N*,*N*,*N*′,*N*′-tetraacetic acid and 5 mM EDTA—at 277 K and with gentle stirring.

### Enzyme activity assay

MsrB1 activity was measured following a published protocol [44] using free methionine sulfoxide (Sigma-Aldrich) as a substrate. Briefly, 100 μl mixture contained 50 mM sodium phosphate, 20 mM NaCl, 20 mM dithiothreitol, 3.5 μM substrate (free methionine sulfoxide), and 5–50 μg purified MsrB1 protein at pH 7.5. The reaction was conducted at 310 K for 75 min. Sampling (100 μl ) was performed every 15 min. The reaction was stopped by adding 200 μl acetonitrile. The concentrations of the substrate (methionine sulfoxide) and the product (methionine) were checked by high-performance liquid chromatography (HPLC).

### Reverse-phase HPLC of methionine residues

The HPLC method used to determine the enzymatic activity of MsrB1 was adapted from that of Minetti et al. [45]. Solvents A and B were used during this procedure. Solvent A was methanol and solvent B was 0.08 M acetate buffer at pH 6.8 with 2 % tetrahydrofuran; the flow rate was 0.9 ml/min at room temperature. The HPLC system comprised a TSP SpectraSYSTEM P2000 gradient pump, a Dionex Ultimate 3000 autoinjector, and a Dionex RF 2000 fluorescence detector. The devices were connected to a computer equipped with the Chromeleon software package. The detector wavelength was set at 436 nm for reading of the absorbance. A Nova Pak LC-18 column (3.9 mm × 150 mm, 4-μm particles) was used for amino acid analysis. *o*-Phthalaldehyde and AAS18 from Sigma were used as a fluorescent reagent and an amino acid standard, respectively.

The following program was used: start with 75 % solvent B for 5 min, then decrease the proportion to 70 % solvent B in 5 min, then decrease the proportion to 50 % solvent B in 5 min, then decrease the proportion to 20 % solvent B in 7 min, and finally decrease the proportion to 0 % solvent B in 3 min. The measurement time was 29 min. Methionine residue was eluted at 19.5 min.

## Results and discussion

### MsrB1-Co expression in M9 culture medium

The *E. coli* ER2566 strain carrying the pET21 His-tagged MsrB1 construct was grown in M9 medium supplemented with Fe^2+^, Mn^2+^, Zn^2+^, and Cu^2+^ ions. The presence of a Zn^2+^ ion within the MsrB1 protein was previously predicted bioinformatically [44] and was proved by our NMR measurements [21]. Zn^2+^ ion is located rather far from the catalytically active residues within the protein and this ion plays a structural role holding together two protein loops in mammalian MsrB1.

**Fig. 1:**
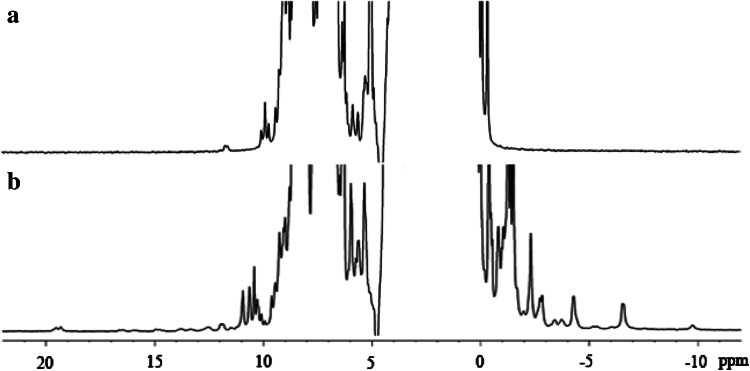
^1^H NMR spectra (600 MHz) of reduced methionine sulfoxide reductase B1 (MsrB1) at 298 K: *a* zinc-containing MsrB1 (MsrB1-Zn); *b* MsrB1 purified from *Escherichia coli* cells grown in M9 medium containing 50 μM Zn^2+^ and 10 μM Co^2+^

Our further studies evidenced that the use of M9 growth medium with the same trace element composition additionally supplemented with Co^2+^ (Co^2+^/Zn^2+^ ratio of 1:5) results in biosynthesis of MsrB1-Co. In an effort to characterize further the process of cobalt insertion into MsrB1, we expressed the protein in M9 medium completely lacking Zn^2+^ ions and in the presence of 10 μM Co^2+^. Surprisingly, even in the full absence of Zn^2+^ ions, cells were able to grow and perform biosynthesis. Thus, Co^2+^ ion has the ability to substitute Zn^2+^ ion both in presence of both metal ions and when the medium contains only Co^2+^ ions. Although the ^1^H NMR spectrum of MsrB1-Zn does not contain any hyperfine-shifted signals (Fig. 1, spectrum a), an insertion of cobalt into MsrB1 was detected by the appearance of the characteristic paramagnetic signals in the ^1^H NMR spectra (Fig. 1, spectrum b). These paramagnetic signals indicate that Co^2+^ ion either competes with Zn^2+^ ion in the metal binding site or binds to an additional metal binding site within the protein. MsrB1 obtained either on repetition of the experiment described or on variation of the Zn^2+^/Co^2+^ ratio (1:5, 1:2, 1:1, 5:1) exhibited an ^1^H NMR spectrum with the paramagnetic signals appearing at exactly the same positions in the spectrum. MsrB1-Co was expressed in the pET21 vector and thus contained six histidines at the protein’s C-terminus that cannot be cleaved since this vector does not contain the thrombin cleavage site. Co^2+^ ion has a strong affinity to histidine residues and, therefore, there was a theoretical possibility that MsrB1 with the His-tag could coordinate Co^2+^ ion. To check this possibility we recloned MsrB1 in the pET28a expression vector, which allows His-tag cleavage. The insertion of cobalt was detected for both MsrB1 with the His-tag and for MsrB1 without the His-tag. In both cases the NMR spectra were identical. This reproducibility unambiguously excludes a possibility of nonspecific binding of Co^2+^ ion to the protein molecule and indicates that insertion of the Co^2+^ ion always follows the same scheme.

### Inhibition of *E. coli* growth by CoCl_2_

**Fig. 2:**
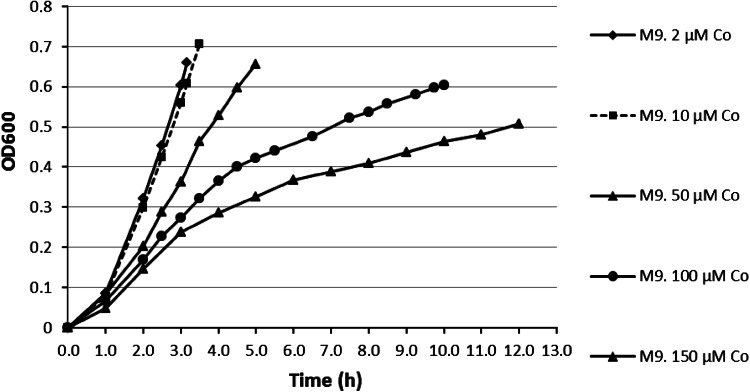
Typical growth curves of *E. coli* in M9 medium containing 10 μM ZnSO_4_ and various concentrations of CoCl_2_ (2, 10, 50, 100, and 150 μM). *OD600* optical density at 600 nm

Cell growth in Zn-containing M9 minimal medium supplemented with CoCl_2_ at different concentrations (2, 10, 50, 100, and 150 μM) is shown in Fig. 2. Homeostasis of cobalt was previously studied for *E. coli* [46]. It was shown that an increase of the Co^2+^ concentration in the growth medium up to 50 μM causes a linear increase of rcnA gene expression. The latter is responsible for Co^2+^ ion efflux from *E. coli* [47]. Therefore, the cell growth inhibition observed in our study is in a good agreement with the literature [46]. Indeed, the presence of cobalt ion begins to inhibit cell growth significantly at a concentration higher than 50 μM.

### Metal affinity for MsrB1-Co and MsrB1-Zn

Our data indicate that removal of metal from MsrB1-Co is pH-dependent and is facilitated by the decrease of pH. Indeed, removal of cobalt from MsrB1-Co was achieved overnight at pH 5.0 and over the course of 6 days at pH 5.5. No cobalt removal was detected at pH 7.0 and 7.5, and no degradation of protein during the process was observed. Removal of zinc was not detected at any pH, confirming previous data that zinc is tightly bound to MsrB1. This observation is in a good agreement with the Irving–Williams series, confirming that zinc is more tightly bound to the protein than is cobalt. Although removal of cobalt was achieved, further reconstitution of MsrB1-Co by a titration of apoprotein with Co^2+^ salt was not detected.

### Electronic UV–visible spectra

**Fig. 3:**
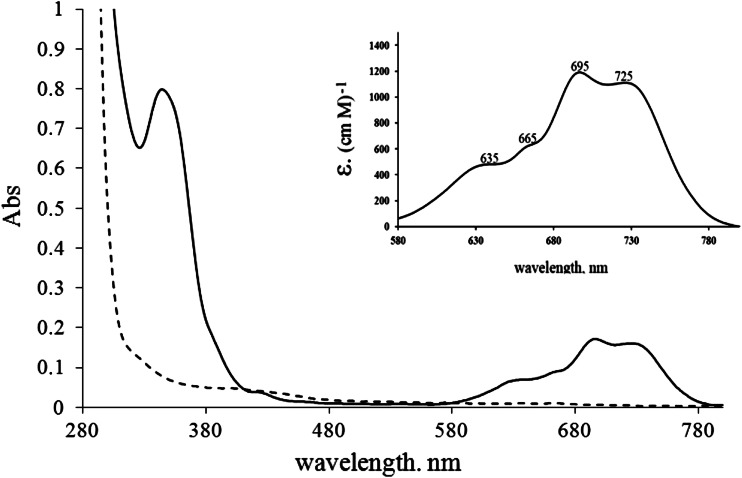
The electron UV–visible absorption spectra of reduced MsrB1 at pH 5.5; *solid line* cobalt-containing MsrB1 (MsrB1-Co) derivative, *dashed line* native MsrB1-Zn. The spectrum of the *d*–*d* transition region of MsrB1-Co expressed in M9 medium completely lacking Zn^2+^ ions, and containing Co^2+^ ions, is shown in the *inset*. Molar extinction coefficient values were derived from the absorbance and protein concentration (10 mg/ml) using the Lambert–Beer law

The electronic UV–visible spectra of MsrB1 overexpressed in *E. coli* in the presence of the Co^2+^ ion exhibit peaks at 345, 635, 665, 695, and 725 nm, which are characteristic of the CysS → Co^2+^ ligand-to-metal charge-transfer band and the *d*–*d* transition bands of Co^2+^, respectively (Fig. 3), and the protein solution is light green-blue. These data further support our finding concerning insertion of Co^2+^ ion into the MsrB1 protein. In addition, the extinction coefficients for the *d*–*d* transition bands (Fig. 3) provided information about the number and the nature of the ligands, as well as the geometry around the metal ion, in particular, suggesting tetrahedral cobalt coordination by four cysteines [48–50].

### Enzyme activity assay

**Fig. 4:**
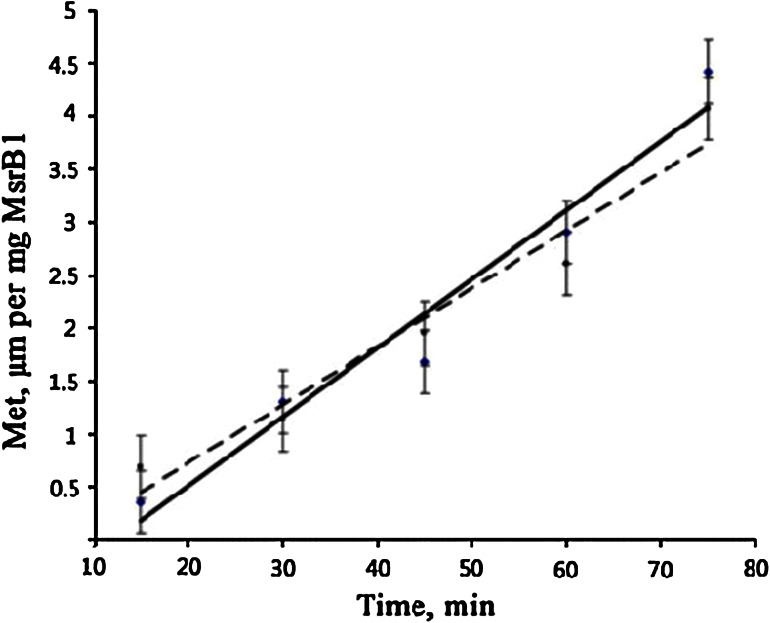
Enzyme activity of MsrB1-Co (*dashed line*) and MsrB1-Zn (*solid line*)

Methionine sulfoxide was reduced to methionine in the presence of MsrB1 and dithiothreitol as a reducing agent. The reaction was linear, for a given amount of protein, up to 75 min incubation. As shown in Fig. 4, MsrB1-Zn and MsrB1-Co exhibit a similar level of activity. These data concerning the functional activities of MsrB1-Zn and MsrB1-Co indicate their structural similarities and very similar catalytic mechanisms. Taking together the activity assay and the UV–visible data, we can conclude that insertion of cobalt into MsrB1 does not perturb the overall fold of the protein, and Co^2+^ is coordinated by the same residues, leaving catalytic cysteines free to exert their function. This conclusion is further supported by the NMR data and the calculations of the magnetic susceptibility anisotropy values reported in subsequent sections.

### One-dimensional ^1^H NMR characterization of MsrB1-Co

**Fig. 5:**
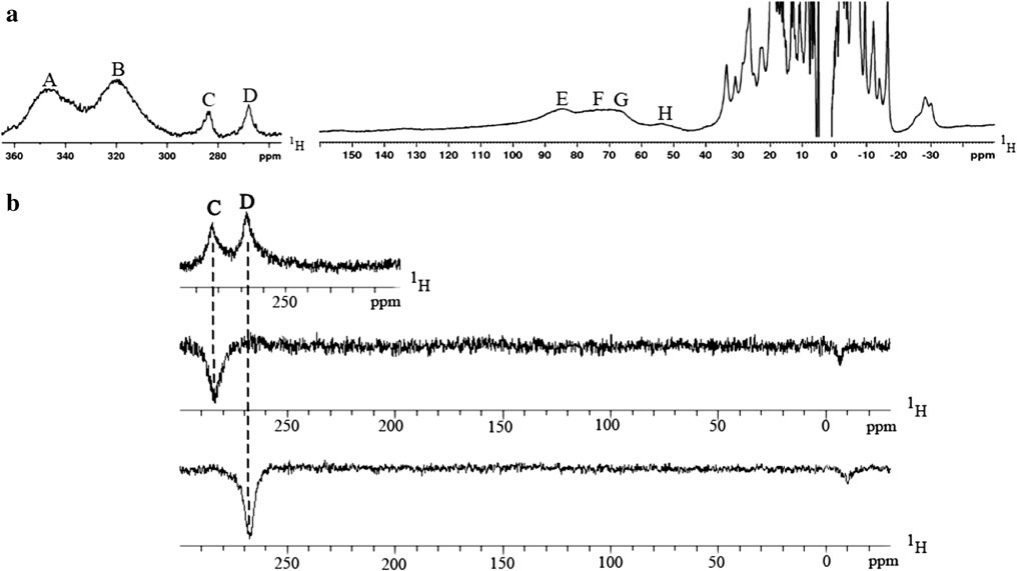
^1^H NMR spectra (600 MHz) of MsrB1-Co recorded with a proton-dedicated probe: **a** full spectrum; **b** one-dimensional nuclear Overhauser effect different spectra obtained on saturation of signals *C* and *D*

As mentioned earlier, the ^1^H NMR spectrum of MsrB1-Co acquired with a standard cryoprobe exhibits several paramagnetically shifted signals, indicating that the protein contains a paramagnetic metal ion. To improve the detection of these paramagnetically influenced NMR signals, the ^1^H NMR spectrum of MsrB1-Co was recorded using a proton-dedicated probe with a 600-MHz spectrometer. An enhanced sensitivity of such probe improves the observation of paramagnetically influenced signals or/and the observation of a higher number of proton signals in the ^1^H NMR spectrum. Indeed, the ^1^H NMR spectrum obtained in this way is characterized by the presence of a significantly higher number of signals spread both upfield and downfield with respect to spectrum b in Fig. 1. The full ^1^H NMR spectrum of MsrB1-Co is reported in Fig. 5a. As can be seen from the spectrum, the paramagnetically shifted signals can be found as far as 350 ppm and they are as broad as 16,000 Hz (signal at 347 ppm), 2,800 Hz (signal at 284 ppm), and 2,700 Hz (signal at 268 ppm). As already known, the paramagnetic Co^2+^ ion has a *d* ^7^ electronic configuration and can be found in either a low-spin state (*S* = 1/2) or a high-spin state (*S* = 3/2). The pattern observed in the ^1^H NMR spectrum of MsrB1-Co (Fig. 5a) and the available literature data [22] suggest that in the case of MsrB1-Co, a high-spin cobalt ion is coordinated to the protein.

In the downfield region of the ^1^H NMR spectrum there are eight signals; these are the broadest in the spectrum and are shifted from 50 to 350 ppm (signals A–H, Fig. 5a). The four cysteines making up the zinc binding site in the native structure remained unassigned in this work (Table S1); thus, it can be supposed that the paramagnetic signals mentioned belong to eight β-CH_2_ protons of four cysteines coordinating cobalt ion as they are the closest to the paramagnetic center. Therefore, the cysteine β-CH_2_ protons should have the maximal contact and dipolar paramagnetic contributions to the chemical shifts and the relaxation values among all MsrB1-Co protons.

The attempts undertaken to find pairwise assignments within these eight broad signals through ^1^H one-dimensional NOE spectra did not result in the observation of any connecting constraints among these signals. However, some NOEs from saturating strongly shifted paramagnetic signals C and D were observed as shown in Fig. 5b. Signals A–H are tentatively assigned by us as β-CH_2_ protons of four coordinating cysteines, whereas the much sharper signals, observed on saturation of signals C and D (Fig. 5b), are tentatively assigned as Hα proton signals of the same cysteine residue to which the saturated signals belong. In addition, the fact that on saturation of the signals C and D the different NOEs are observed indicates that the signals C and D belong to the different cysteines coordinating Co^2+^ ion.

### Heteronuclear NMR assignment of MsrB1-Co

Two-dimensional and three-dimensional NMR spectra of MsrB1-Co were recorded and subsequent backbone and side-chain resonance assignments were performed. The results are reported in Table S1. About 70 % of backbone nuclei were assigned through this procedure (excluding prolines and the His-tag). Side-chain assignments was accomplished using the HCCH-TOCSY of MsrB1-Co. As a result, the side-chain assignment obtained covered 62 out of the 79 residues for which the backbone assignment was achieved. This constitutes 57 % of the entire protein. Taking into consideration a rather strong paramagnetic property of the system, this result by itself represents a significant achievement.

### Comparison of chemical shift assignments for MsrB1-Zn and MsrB1-Co

Comparison of the ^1^H and ^15^N resonances of pure MsrB1-Zn and MsrB1-Co showed that generally almost all NH and N resonances within the protein changed their chemical shifts on passing from MsrB1-Zn to MsrB1-Co.

**Fig. 6:**
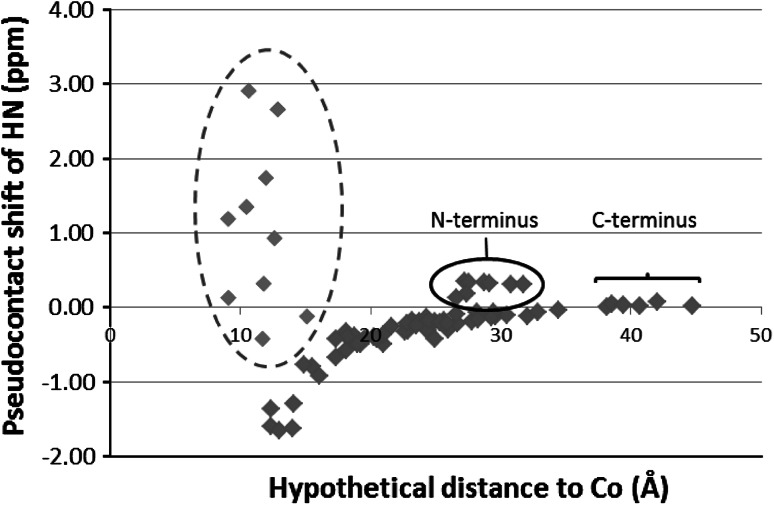
The paramagnetic pseudocontact shift contribution versus the distance from the NH group of each MsrB1 residue to the metal ion. The reported distances were obtained from the third conformer of MsrB1. The *dashed-line ellipse* indicates the residues which do not obey to the general trend. The *solid-line ellipse* indicates N-terminal residues

This observation may indicate either that the structure of MsrB1-Zn completely changes on Co^2+^ for Zn^2+^ substitution or that the paramagnetic Co^2+^ ion strongly affects all chemical shifts within MsrB1 while the structure of the protein itself may remain almost invariable. The chemical shift comparison data are shown in Fig. 6, where the observed differences in chemical shift of amide protons are reported as a function of their distance from Co^2+^ ion (it was supposed that the structure of MsrB1-Co remains almost identical to that of MsrB1-Zn, and Co^2+^ ion takes the same position as Zn^2+^ ion in the MsrB1 protein). As it can be seen from Fig. 6, as the proton–cobalt distances within MsrB1 increase, the chemical shift difference decreases to zero. This behavior is in agreement with the expected paramagnetic pseudocontact shift contribution to the observed chemical shift, which is inversely related to the third power of the distance between the nucleus and the paramagnetic center [22].

However, despite the observed good “chemical shift difference–distance” agreement, ten residues (Thr49, Ile50, His51, Asp53, Ser54, Val55, Phe97, Ser99, Ser100, and Leu101) are shown to diverge from the general behavior in all MsrB1 family conformers (PDB ID 2KV1) inspected (Fig. 6, in the dashed-line ellipse). Further investigation of the location of these residues in MsrB1-Zn revealed that the residues mentioned above constitute two unstructured loops in the vicinity of the metal ion. Thus, the observed deviations from the expected chemical shifts might be explained by the increased mobility of the loops mentioned with respect to the rest of the protein. This mobility remained unobserved for diamagnetic MsrB1-Zn.

In addition, another group of signals belonging to the residues of the flexible N-terminal part (Fig. 6, in the solid-line ellipse) was found to deviate from the general trend. At the same time, residues belonging to the C-terminal flexible region have pseudocontact shifts of zero. Thus, the small positive pseudocontact shifts observed for the N-terminal amino acids indicate that the N-terminus is positioned on average closer to the MsrB1’s metal and catalytic centers than expected from the previously reported protein structure and it spends a significant amount of time near the metal binding site (Co^2+^ ion in the present case) [21]. As MsrB1 activity depends on the formation of an intramolecular disulfide bridge between the catalytic Sec/cysteine and the resolving cysteine (the MsrB1 N-terminal part), it would not be surprising that the N-terminus tends towards proximity to the catalytic residue and spends time there on the order of the millisecond range as indicated by our NMR data. The latter should result in increase of the protein’s catalytic efficiency. Therefore, our NMR data provide further indirect support for the earlier proposed catalytic mechanism of MsrB1 [13].

**Fig. 7:**
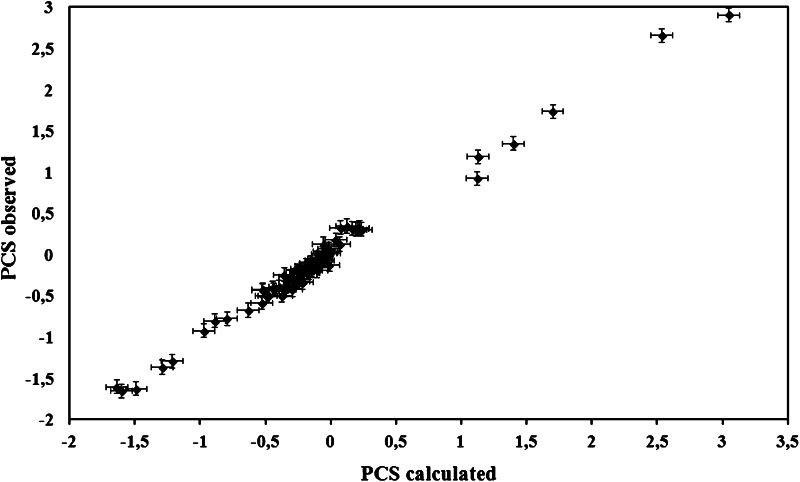
The best correlation between the observed and calculated pseudocontact shifts (*PCS*) belonging to the third conformer of MsrB1-Zn

The observed “differences in chemical shift versus the distance to metal ion” dependencies were further analyzed using structural minimization. For this purpose, 77 experimental pseudocontact shifts and 20 available conformers of the structural family of MsrB1-Zn were used as an input to calculate the minimized structure of MsrB1-Co using the program Amber. All the calculated structures exhibited close similarity to the original MsrB1-Zn structural conformers. However, the best correlation between the observed and calculated pseudocontact shifts was observed for the family’s third conformer (Fig. 7); therefore, this conformer was selected for further structural analysis.

The data concerning the metal binding site of MsrB1-Co obtained from UV–visible spectroscopy are in a good agreement with the Amber calculations. Indeed, the UV–visible spectra (Fig. 3) indicate that in MsrB1-Co the metal ion has a tetrahedral geometry and is coordinated by four cysteines.

**Fig. 8:**
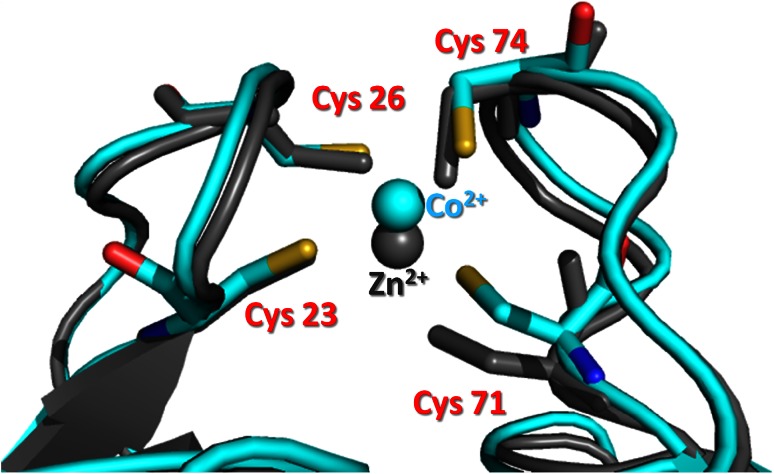
Superimposition of the active sites of MsrB1-Zn (*gray*) and calculated MsrB1-Co (*cyan*)

The metal coordination sites of MsrB1-Zn and MsrB1-Co are compared in Fig. 8. Cobalt binding site exhibits little reorientation of the side chain of the coordinating cysteines and the metal ion. However, the variations of the Cβ and Sγ atoms of coordinating cysteines Cys71, Cys74, and Cys23 (move by 1.2, 0.4, and 0.5 Å for Cβ and 1.6, 0.9, and 0.5 Å for Sγ, respectively) with respect to the MsrB1-Zn protein are within the root mean square deviation reported for the side chains (1.7 ± 0.31 Å) of the MsrB1-Zn family [51]. The backbone, in contrast, does not exhibit significant variations between the two structures. Indeed, the maximal difference for Cα of the coordinating cysteines is 0.8 Å (for Cys71), which is also below the root mean square deviation reported for the backbone atoms (1.59 ± 0.47 Å) in the MsrB1-Zn family structure [21].

### Calculation of magnetic susceptibility anisotropy values

The AnisoFit calculation performed on the minimized MsrB1-Co structure drastically improved the results with respect to the not-minimized structure. Indeed, the magnetic susceptibility anisotropy values (∆*χ*_ax_ = 15.19 × 10^−32^ m^3^ and ∆*χ*_rh_ = −0.97 × 10^−32^ m^3^) permitted us to obtain calculated pseudocontact shifts that are in a very good agreement with the experimental values (Fig. 7).

**Fig. 9:**
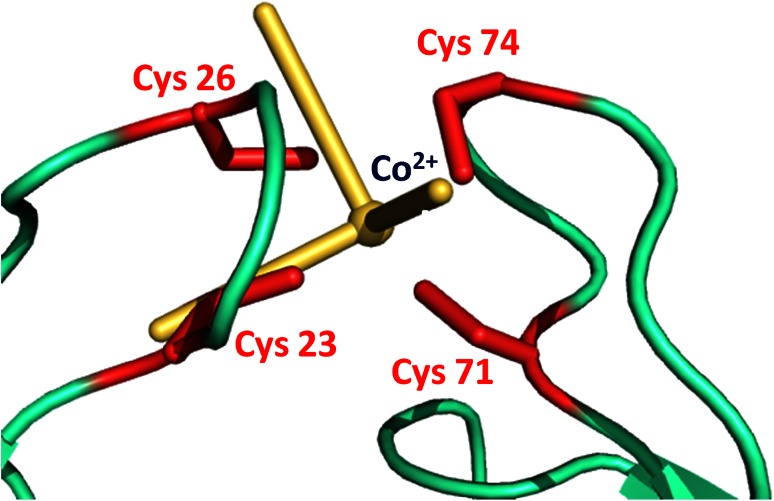
The magnetic susceptibility tensor orientation centered on Co^2+^ ion (*yellow*) determined from the experimental pseudocontact shifts using the third conformer of MsrB1 (PDB ID 2KV1) structure family. Cysteines coordinating the metal ion are reported in *red*

Figure 9 shows a metal binding site of the minimized MsrB1-Co with the axial and rhombic anisotropy parameters of the magnetic susceptibility tensor centered on Co^2+^ ion. The tensor is characterized by the *xy*-plane, with one axis pointing towards Cys23(Sγ), with an axis-Co–Cys 23(Sγ) angle of about 22°, and a second axis pointing towards the Cys26(Sγ), with an axis-Co–Cys26(Sγ) angle of about 36°. The *z*-axis is situated approximately along a molecular *C*_2_ axis of symmetry of the tetrahedral coordination geometry of the ion, passing between Cys23(Sγ) and Cys74(Sγ). The magnetic susceptibility anisotropy values (∆*χ*_ax_ = 15.19 × 10^−32^ m^3^ and ∆*χ*_rh_ = −0.97 × 10^−32^ m^3^) reveal a prevalent axial component which is in accordance with a disordered tetrahedral high-spin Co^2+^ ion coordination reported for other systems such as cobalt-substituted rubredoxin and cobalt-substituted desulforedoxin [52].

### Methodological use of the proposed approach

Zinc is one of the most important biological metals [1, 2, 53, 54]. The ability to determine the primary protein structure through the translation of DNA sequences now allows the prediction of zinc binding sites and, thereby, the enzyme function [9]. As mentioned already, it is possible to distinguish three types of zinc binding sites: catalytic, co-catalytic, and structural. Zinc metal sites found both in mammalian and in bacterial MsrB proteins play a structural role. Zn^2+^ ion in MsrB proteins is usually coordinated by cysteine residues. In the proteins in which the alignment shows an absence of cysteine residues (substituted by Asp, Thr, Ser, Gly, and Ala), it was bioinformatically predicted that the corresponding MsrB proteins lack a Zn^2+^ ion. In addition, the X-ray structural analysis of such MsrB proteins shows the absence of a Zn^2+^ ion in the structures determined. However, the analysis of the crystallization conditions for the published structures of MsrB proteins where Zn^2+^ ion is absent (no “classic” zinc binding CXXC motif) indicates that crystallization occurred in mild acidic environments [55–57]. Some of the “nonclassic” Zn^2+^ ligands can be protonated (influenced by the solution pH) under these conditions. In other words, when Zn^2+^ ligands are protonated, they are not able to coordinate the metal ion. In addition, the reported absence of a spectroscopically silent Zn^2+^ ion for some experimentally characterized MsrB proteins may simply be because the metal ion is lost during isolation and purification of the protein since the coordination bonds between Zn^2+^ and an oxygen-containing ligand are weaker with respect to the usual cysteine ligand. Thus, Zn^2+^ was not present in such measurements. The structural similarities within metal-coordinating loops among all MsrB proteins would indirectly support this suggestion.

The approach reported in this article demonstrates that cysteine-containing MsrB1 expressed in *E. coli* in the cobalt-containing M9 medium can replace Zn^2+^ ion with Co^2+^. Insertion of Co^2+^ ion can be easily detected by both UV–visible spectroscopy (characteristic light-blue color) and NMR spectroscopy (the appearance of paramagnetically shifted signals). It is known [1, 58] that Co^2+^ ion can be used to substitute Zn^2+^ ion since both metal ions have similar radii and coordination properties. Thus, the technique proposed by us is an easy method to check and confirm whether Zn^2+^ binds only four cysteines and whether no other ligands at different, more basic pH can coordinate the metal ion.

## Conclusions

The study performed involved a phenomenon of Co^2+^ for Zn^2+^ substitution in a mammalian protein, MsrB1, under expression in prokaryote cells (*E. coli*). It was shown that the expression of MsrB1 in cobalt-containing medium resulted in the reproducible appearance of the new protein—MsrB1-Co. Blue MsrB1-Co was found to be stable with time, and exhibited *d*–*d* transitions at wavelengths and with extinction coefficients characteristic of tetrahedral coordination geometry.

^1^H NMR spectra of the protein revealed along with a good signal dispersion (indicative that the protein is folded) the presence of strongly paramagnetic signals from −30 to 350 ppm, as well as their significant broadening due to insertion of Co^2+^ ion into MsrB1. Eight signals were tentatively attributed to β-CH_2_ atoms of cysteines coordinating Co^2+^ ion.

Further NMR studies of MsrB1-Co allowed us to make 70 % of backbone and 57 % of side-chain assignments. The AnisoFit and Amber calculations based on the native form of MsrB1 and the experimentally obtained pseudocontact shifts allowed us to generate a structure of MsrB1-Co which was found to share the overall fold with MsrB1-Zn. Further analysis suggested that the N-terminus is not fully disordered as follows from the MsrB1-Zn structure but is rather in conformational exchange on the millisecond timescale and spends a significant amount of time near the metal binding site. This proximity ensures high catalytic efficiency because of the short distance between the catalytic and resolving residues.

Functional studies showed that both MsrB1-Zn and MsrB1-Co exhibit similar levels of activity in agreement with the structural similarity found.

A methodological use of the proposed approach for substitution of Zn^2+^ ion by Co^2+^ ion was proposed to monitor whether other MsrB proteins contain a metal ion.

## Electronic supplementary material

Below is the link to the electronic supplementary material.

**Table S1** Assigned NMR chemical shifts of MsrB1-Co (*T* = 298 K) (PDF 47 kb)

## Abbreviations

HPLC: High-performance liquid chromatography
HSQC: Heteronuclear single-quantum correlation
Msr: Methionine sulfoxide reductase
MsrB1: Methionine sulfoxide reductase B1
MsrB1-Co: Cobalt-containing methionine sulfoxide reductase B1
MsrB1-Zn: Zinc-containing methionine sulfoxide reductase B1
NMR: Nuclear magnetic resonance
NOE: Nuclear Overhauser effect
PDB: Protein Data Bank
Sec: Selenocysteine

## Footnotes

1 AnisoFit is available online at http://py-enmr.cerm.unifi.it/access/index/anisofit.
