# Supplementary material for: Competitive cobalt for zinc substitution in mammalian methionine sulfoxide reductase B1 overexpressed in E. coli: structural and functional insight
Source: J Biol Inorg Chem. 2013 Nov 24;19(1):85–95. doi: 10.1007/s00775-013-1064-7 (PMC3889830; doi:10.1007/s00775-013-1064-7)
Supplement: Supplementary file 1 — Table S1 Assigned NMR chemical shifts of MsrB1-Co (T = 298 K) (PDF 47 kb) [file 775_2013_1064_MOESM1_ESM.pdf]

Table S1. Assigned chemical shifts of MsrB1-Co (T=298K).

Residue	Atom	Chemical shift (ppm)	Residue	Atom	Chemical shift (ppm)	Residue	Atom	Chemical shift (ppm)
4	CA	55.833	11	CG2	15.947	19	HA3	4.007
4	CB	25.507	11	H	8.073	19	N	110.016
4	H	8.513	11	HA	3.404	20	CA	56.263
4	HA	4.338	11	HB	1.430	20	CB	32.348
4	N	120.927	11	HG1	0.235	20	H	7.145
5	C	171.122	11	HG2	-0.032	20	HA	2.905
5	CA	55.801	11	N	119.542	20	HB	0.651
5	CB	60.912	12	CA	52.840	20	HG1	-1.465
5	H	8.565	12	CB	34.543	20	N	119.070
5	HA	4.372	12	H	7.543	21	CA	51.397
5	HB2	3.804	12	HA	4.605	21	CB	33.187
5	HB3	3.762	12	N	112.618	21	H	7.965
5	N	119.018	13	CA	59.245	21	HA	2.332
6	C	172.574	13	CB	23.726	21	HB2	0.244
6	CA	55.336	13	CG	29.389	21	HB3	-0.501
6	CB	36.843	13	H	7.896	21	N	120.535
6	H	8.335	13	HA	2.751	22	H	6.418
6	HA	4.641	13	HG2	1.896	35	CA	55.265
6	HB2	3.052	13	HG3	1.896	35	CB	61.921
6	HB3	2.980	13	N	125.419	35	H	7.631
6	N	122.045	14	CA	48.526	35	HA	4.363
7	C	173.236	14	CB	34.468	35	HB2	3.805
7	CA	55.125	14	H	8.398	35	HB3	3.765
7	CB	36.720	14	HA	4.606	35	N	113.441
7	H	8.548	14	HB2	2.602	36	CA	52.449
7	HA	4.672	14	HB3	2.300	36	CB	32.175
7	HB2	3.137	14	N	114.860	36	H	7.246
7	HB3	3.053	15	CA	53.675	36	HA	4.162
7	N	122.532	15	CB	30.009	36	HB2	0.712
8	C	171.682	15	H	5.744	36	HB3	0.712
8	CA	42.931	15	HA	3.798	36	N	125.743
8	H	8.197	15	HB2	2.523	37	CA	52.460
8	HA2	4.605	15	HB3	2.523	37	CB	37.669
8	HA3	4.605	15	N	119.424	37	H	8.051
8	N	110.741	16	C	171.305	37	HA	4.679
9	C	172.562	16	CA	54.119	37	HB2	2.501
9	CA	42.604	16	CB	36.071	37	HB3	2.501
9	H	8.258	16	H	8.582	37	N	123.012
9	N	107.860	16	HA	4.222	38	CA	49.417
10	C	175.503	16	HB2	3.169	38	CB	14.855
10	CA	52.092	16	HB3	3.169	38	H	8.396
10	CB	26.371	16	N	126.185	38	HA	4.197
10	H	8.583	17	CA	50.435	38	HB	0.808
10	HA	4.403	17	CB	27.181	38	N	124.069
10	HB2	1.816	17	H	5.358	39	CA	53.537
10	HB3	1.816	17	HA	3.512	39	CB	31.200
10	N	122.110	17	N	118.776	39	H	7.880
11	CA	61.298	19	CA	42.676	39	HA	4.202
11	CB	28.123	19	H	8.393	39	HB2	2.804
11	CG1	17.311	19	HA2	4.007	39	HB3	1.964

Residue	Atom	Chemical shift (ppm)	Residue	Atom	Chemical shift (ppm)	Residue	Atom	Chemical shift (ppm)
39	N	124.756	48	HA	4.349	57	CA	50.323
40	CA	56.022	48	HB2	2.061	57	CB	31.792
40	CB	59.673	48	HB3	1.749	57	H	7.249
40	H	6.737	48	N	114.197	57	HA	3.483
40	HA	3.763	49	CA	57.173	57	N	119.350
40	HB2	3.290	49	CB	67.438	58	CA	51.383
40	HB3	3.290	49	CG2	18.789	58	CB	25.255
40	N	117.760	49	H	8.034	58	H	7.316
41	CA	54.977	49	HA	3.551	58	HA	3.950
41	CB	60.309	49	HB	4.265	58	HB2	1.449
41	H	9.043	49	HG2	-0.591	58	HB3	1.449
41	HA	4.196	49	N	110.231	58	N	118.691
41	HB2	3.654	50	CA	62.084	61	C	173.948
41	HB3	3.654	50	CB	36.307	61	CA	55.252
41	N	121.652	50	CD1	12.276	61	CB	28.359
43	CA	54.297	50	H	8.039	61	CG	21.342
43	CB	26.562	50	HA	3.916	61	CD	29.389
43	H	6.454	50	HB	1.385	61	CE	38.909
43	HA	4.423	50	HG1	1.093	61	H	8.521
43	HB2	2.459	50	HD1	0.649	61	HA	3.505
43	HB3	2.459	50	N	121.420	61	HB2	1.288
43	N	112.992	51	CA	51.242	61	HB3	1.288
45	CA	46.716	51	CB	29.853	61	HG2	1.025
45	CB	17.403	51	H	9.139	61	HG3	1.000
45	H	7.363	51	HA	6.606	61	HD2	1.684
45	HA	4.444	51	HB2	5.759	61	HD3	1.684
45	HB	0.070	51	HB3	5.759	61	HE2	2.596
45	N	125.222	51	N	116.500	61	HE3	2.596
46	CA	51.219	53	CA	52.210	61	N	126.064
46	CB	40.449	53	CB	39.874	62	C	171.357
46	H	7.414	53	H	10.727	62	CA	50.945
46	HA	5.086	53	HA	6.712	62	CB	34.763
46	HB2	2.204	53	HB2	6.305	62	H	8.697
46	HB3	1.012	53	HB3	6.305	62	HA	4.166
46	N	114.069	53	N	116.000	62	HB2	2.605
47	CA	59.945	54	CA	66.671	62	HB3	2.499
47	CB	67.201	54	CB	64.834	62	N	115.939
47	CG2	21.062	54	H	10.633	63	C	169.161
47	H	8.786	54	HA	6.672	63	CA	50.793
47	HA	4.648	54	N	119.522	63	CB	27.989
47	HB	4.071	55	CA	53.969	63	H	7.207
47	HG2	0.794	55	CB	29.296	63	HA	4.398
47	N	108.570	55	H	8.549	63	HB2	1.351
48	CA	52.174	55	HA	2.641	63	HB3	1.032
48	CB	28.927	55	HB	0.158	63	N	118.151
48	H	6.899	55	N	105.494	65	C	171.195

Residue	Atom	Chemical shift (ppm)	Residue	Atom	Chemical shift (ppm)	Residue	Atom	Chemical shift (ppm)
65	CA	53.691	85	CA	50.782	90	H	8.875
65	CB	25.017	85	CB	42.142	90	HA2	3.933
65	H	9.034	85	H	6.920	90	HA3	3.510
65	HA	3.818	85	HA	4.818	90	N	114.322
65	HB2	1.762	85	HB2	2.030	91	C	173.937
65	HB3	1.762	85	HB3	1.773	91	CA	53.048
65	N	116.066	85	N	116.210	91	CB	28.197
66	C	171.561	86	C	168.644	91	H	8.031
66	CA	47.172	86	CA	41.546	91	HA	4.202
66	CB	18.480	86	H	8.041	91	HB2	1.772
66	H	7.731	86	HA2	4.814	91	HB3	1.772
66	HA	4.695	86	HA3	4.814	91	N	120.064
66	HB	0.405	86	N	106.576	92	C	168.609
66	N	123.605	88	C	172.649	92	CA	54.548
67	C	173.212	88	CA	50.902	92	CB	62.049
67	CA	49.500	88	CB	31.820	92	H	8.996
67	CB	39.440	88	CG	21.857	92	HA	4.272
67	H	8.217	88	CD	26.404	92	HB2	2.897
67	HA	3.855	88	CE	38.679	92	HB3	2.897
67	N	119.046	88	H	7.207	92	N	119.888
82	CA	53.676	88	HA	4.188	93	C	172.857
82	CB	35.302	88	HB2	1.418	93	CA	51.974
82	H	8.650	88	HB3	1.141	93	CB	31.681
82	HA	4.041	88	HG2	0.980	93	H	7.451
82	N	125.630	88	HG3	0.980	93	HA	4.290
83	CA	52.558	88	HD2	1.317	93	N	116.603
83	CB	38.125	88	HD3	1.292	94	C	169.532
83	CD1	21.682	88	HE2	2.640	94	CA	54.834
83	CD2	19.302	88	HE3	2.572	94	CB	34.488
83	H	7.946	88	N	115.263	94	H	9.276
83	HA	3.623	89	C	174.616	94	HA	3.688
83	HB2	1.147	89	CA	55.208	94	HB2	1.814
83	HB3	0.918	89	CB	26.224	94	HB3	0.939
83	HG	2.570	89	CG	23.449	94	N	125.639
83	HD1	0.327	89	CD	40.384	95	C	169.538
83	HD2	0.112	89	H	8.263	95	CA	54.119
83	N	125.612	89	HA	3.679	95	CB	25.092
84	C	171.505	89	HB2	1.443	95	H	8.392
84	CA	52.153	89	HB3	1.443	95	HA	3.898
84	CB	34.651	89	HG2	1.321	95	N	126.799
84	H	9.061	89	HG3	1.321	96	C	170.432
84	HA	3.917	89	HD2	2.937	96	CA	52.050
84	HB2	2.683	89	HD3	2.937	96	CB	35.281
84	HB3	2.439	89	N	121.310	96	CG1	21.062
84	N	115.885	90	C	171.970	96	H	6.740
85	C	172.935	90	CA	42.123	96	HA	3.460

Residue	Atom	Chemical shift (ppm)	Residue	Atom	Chemical shift (ppm)	Residue	Atom	Chemical shift (ppm)
96	HB	0.127	102	HB3	-2.504	108	CA	53.166
96	HG1	-0.303	102	N	128.768	108	CB	29.584
96	HG1	-1.733	103	C	169.440	108	CG	21.569
96	HG2	-1.207	103	CA	52.089	108	CD	29.105
96	N	128.960	103	CB	37.022	108	CE	39.022
97	C	176.010	103	H	7.363	108	H	8.024
97	CA	54.775	103	HA	2.588	108	HA	3.874
97	CB	35.738	103	HB2	1.351	108	HB2	1.235
97	H	7.623	103	HB3	0.776	108	HB3	1.207
97	HA	4.774	103	N	121.711	108	HG2	0.939
97	HB2	3.573	104	C	168.964	108	HG3	0.939
97	HB3	3.536	104	CA	54.612	108	HD2	1.351
97	N	121.144	104	CB	27.989	108	HD3	1.351
98	CA	62.272	104	CG1	16.402	108	HE2	2.536
98	CB	60.684	104	H	6.934	108	HE3	2.536
98	H	11.846	104	HA	2.642	108	N	120.346
98	N	126.436	104	HB	0.173	109	C	173.394
99	C	175.582	104	HG1	-0.595	109	CA	53.664
99	CA	58.939	104	HG2	-1.725	109	CB	26.952
99	CB	61.435	104	N	129.551	109	CG	33.217
99	H	10.289	106	C	173.641	109	H	8.488
99	HA	5.497	106	CA	53.702	109	HA	3.753
99	HB2	6.122	106	CB	30.235	109	HB2	1.655
99	HB3	6.122	106	CG	21.517	109	HB3	1.562
99	N	114.182	106	CD	26.176	109	HG2	1.909
100	CA	60.947	106	CE	39.020	109	HG3	1.909
100	CB	63.940	106	H	7.475	109	N	121.711
100	H	9.496	106	HA	3.618	110	C	174.717
100	HA	-3.185	106	HB2	1.171	110	CA	49.650
100	N	120.285	106	HB3	0.972	110	CB	16.196
101	C	171.122	106	HG2	0.936	110	H	8.259
101	CA	48.431	106	HG3	0.936	110	HA	3.829
101	CB	39.929	106	HD2	1.249	110	HB	0.950
101	H	8.235	106	HD3	1.249	110	N	125.163
101	HA	4.183	106	HE2	2.565	111	C	174.876
101	HB2	1.902	106	HE3	2.565	111	CA	49.721
101	HB3	1.427	106	N	121.966	111	CB	16.181
101	N	120.834	107	C	170.962	111	H	8.119
102	C	168.855	107	CA	41.963	111	HA	3.900
102	CA	49.683	107	H	8.505	111	HB	1.022
102	CB	30.128	107	HA2	3.480	111	N	122.808
102	H	6.206	107	HA3	3.480	112	C	175.003
102	HA	2.652	107	N	112.483	112	CA	49.801
102	HB2	-1.972	108	C	173.686	112	CB	15.797

Residue	Atom	Chemical shift (ppm)	Residue	Atom	Chemical shift (ppm)	Residue	Atom	Chemical shift (ppm)
112	H	8.147	115	C	171.076	117	HD1	0.659
112	HA	3.957	115	CA	42.738	117	HD2	0.601
112	HB	1.079	115	H	8.414	117	N	123.276
112	N	122.760	115	HA2	3.692	118	C	173.286
113	C	171.891	115	HA3	3.692	118	CA	53.714
113	CA	55.669	115	N	109.315	118	CB	27.405
113	CB	60.804	116	C	172.006	118	CG	33.244
113	H	8.159	116	CA	52.933	118	H	8.417
113	HA	4.111	116	CB	26.556	118	HA	3.973
113	HB2	3.613	116	H	8.312	118	HB2	1.709
113	HB3	3.566	116	HA	4.398	118	HB3	1.662
113	N	114.573	116	HB2	2.910	118	HG2	2.011
114	C	173.651	116	HB3	2.910	118	HG3	1.959
114	CA	53.172	116	N	118.453	118	N	121.451
114	CB	26.499	117	C	174.216	119	C	171.744
114	CG	33.242	117	CA	52.362	119	CA	52.935
114	H	8.331	117	CB	39.406	119	CB	26.919
114	HA	3.992	117	CD1	21.403	119	H	8.430
114	HB2	1.856	117	CD2	52.362	119	HA	4.430
114	HB3	1.660	117	H	8.247	119	HB2	2.831
114	HG2	2.008	117	HA	4.083	119	HB3	2.831
114	HG3	1.949	117	HB2	1.196	119	N	119.821
114	N	121.621	117	HB3	1.196			
